# Metatranscriptome analysis of the microbial fermentation of dietary milk proteins in the murine gut

**DOI:** 10.1371/journal.pone.0194066

**Published:** 2018-04-17

**Authors:** Floor Hugenholtz, Mark Davids, Jessica Schwarz, Michael Müller, Daniel Tomé, Peter Schaap, Guido J. E. J. Hooiveld, Hauke Smidt, Michiel Kleerebezem

**Affiliations:** 1 Laboratory of Microbiology, Wageningen University, Wageningen, The Netherlands; 2 Netherlands Consortium for Systems Biology, Amsterdam, The Netherlands; 3 Laboratory of Systems and Synthetic Biology, Wageningen University, Wageningen, The Netherlands; 4 Nutrition, Metabolism and Genomics Group, Wageningen University, Wageningen, The Netherlands; 5 AgroParisTech, INRA, UMR914 Nutrition Physiology and Ingestive Behavior, Paris, France; 6 TI Food and Nutrition, Wageningen, The Netherlands; 7 Host Microbe Interactomics Group, Wageningen University, Wageningen, The Netherlands; Southern Illinois University School of Medicine, UNITED STATES

## Abstract

Undigestible food ingredients are converted by the microbiota into a large range of metabolites, predominated by short chain fatty acids (SCFA). These microbial metabolites are subsequently available for absorption by the host mucosa and can serve as an energy source. Amino acids fermentation by the microbiota expands the spectrum of fermentation end-products beyond acetate, propionate and butyrate, to include in particular branched-SCFA. Here the long-term effects of high protein-diets on microbial community composition and functionality in mice were analyzed. Determinations of the microbiota composition using phylogenetic microarray (MITChip) technology were complemented with metatranscriptome and SCFA analyses to obtain insight in in situ expression of protein fermentation pathways and the phylogenetic groups involved. High protein diets led to increased luminal concentrations of branched-SCFA, in accordance with protein fermentation in the gut. Bacteria dominantly participating in protein catabolism belonged to the *Lachnospiraceae*, *Erysipelotrichaceae* and *Clostridiaceae* families in both normal- and high- protein diet regimes. This study identifies the microbial groups involved in protein catabolism in the intestine and underpins the value of in situ metatranscriptome analyses as an approach to decipher locally active metabolic networks and pathways as a function of the dietary regime, as well as the phylogeny of the microorganisms executing them.

## Introduction

Components of our daily food such as fibers and a part of our dietary protein are not efficiently digested and absorbed in the small intestine. These food ingredients proceed toward the large intestine where they are converted by the microbiota into a large range of metabolites, of which short chain fatty acids (SCFA) are the most abundant. These microbial metabolites are subsequently available for absorption by the host mucosa and can serve as an energy source.

Approximately ten grams of protein reach the human colon daily [1], which include both host (pancreatic enzymes and mucins) as well as dietary proteins. The gut microbiota has a high proteolytic capacity and ferments the proteins into SCFA, branched chain fatty acids (BCFA), ammonia and phenolic and indolic compounds [2–5]. These BCFA are generated by branched-chain amino acid catabolism, i.e., the degradation of valine, leucine and isoleucine [6], while the phenolic and indolic compounds are degradation products of aromatic amino acids.

Nowadays, the consumption of diets that contain high protein levels is quite common and has been proposed to support body weight reduction, where both fat or carbohydrates replacement by protein results in weight loss [7–10]. With respect to the effect of high protein dietary intake on the composition of the gut microbiota, it has been shown that long term consumption of high levels of protein and animal fat are associated with the *Bacteroides* enterotype [11,12], but it should be noted that so far there is no confirmation that the high protein content in the diets directly leads to this enterotype. In rats and mice a short term (2 weeks) high protein dietary intervention did change the microbial community, where the relative abundance of *Clostridium coccoides* (*Lachnospiraceae)* and *Clostridium leptum* groups (*Ruminococcaceae)* and *Faecalibacterium prausnitzii* (*Ruminococcaceae)* decreased due to the intervention [13,14]. Longer term, up to six weeks, high protein diets also induced microbiota shifts in rats, where butyrate producing species belonging to *Lachnospiraceae* and *Ruminococcaceae* decreased and *Escherichia coli* increased [15]. With very long term high-protein/high-fat dietary intervention, 16 month in mice, the microbiota shift maintains and results in similar survival then a low fat diet [16]. However, to date little is known about the effect of this type of high-protein diets and the microbiota shifts on the bacterial fermentation of protein by the gut microbiota.

In a previous study the effect of long-term high protein (HP) diets was studied in a mouse model, and could be shown to result in a lower body weight, reduced adiposity and hepatic lipid accumulation [17]. Here we describe the effects of long-term high-protein dietary interventions on gut microbiota composition and activity in mice, using 16S ribosomal RNA (rRNA) gene-targeted community analysis and metatranscriptome profiling approaches, to unravel patterns of activity within the murine caecal microbial ecosystem.

## Methods

### Ethics statement

All animal experiments were approved by the Animal Experimentation Board at Wageningen University (record #2010017) and carried out according to the guidelines of the European Convention of Vertebrate Animals Used for Experimentation, under European Council Directive 86/609/EEC dated November, 1986.

### Mice and diets

The animals used in this study are previously described by Schwarz and co-authors **[17]**. Male C57BL/6J mice (age 8 weeks) were purchased from Charles River (L’Arbresle, France) and were housed in the animal facility of the Wageningen University. The mice were divided into four groups of 10 animals and housed in pairs in light and temperature-controlled animal housing facilities (12/12 (light/dark), 20°C). The mice had free access to food and tap water. During the first two weeks of the study all mice received the same diet, containing (in %w/totalw) casein (14), corn starch (36.1), sucrose (36.1), soy oil (4), mineral mixture (3.5), vitamin mixture (1), cellulose (5) and choline (0.23). This control diet (NPLF) was given to one group of mice during the whole experiment. In the other groups the amount of protein, fat and carbohydrates was changed (Table 1). The responses of the mice to the dietary interventions was reported previously [17]. The mice were sacrificed after 12 weeks of dietary intervention and were anaesthetised with Isoflurane, the intestinal content was collected from the ileum, caecum and colon and snap frozen in liquid nitrogen and stored at -80°C.

**Table 1:** The composition of ingredients in the four diets. NPLF: Normal protein low fat. NPHF Normal protein high fat. HPLF: High protein low fat. HPHF: high protein high fat [17].

	NPLF	NPHF	HPLF	HPHF
	(g/kg) dry matter			
**Milk protein**	140	160	484	580
**Corn starch**	361.35	291.3	189.35	80
**Sucrose**	361.35	291.4	189.35	80
**Soybean oil**	40	40	40	40
**Palm oil**	0	120	0	123
**Minerals**	35	35	35	35
**Vitamins**	10	10	10	10
**a-Cellulose**	50	50	50	50
**Choline**	2.3	2.3	2.3	2.3

### Short-chain fatty acid analysis in caecal luminal content

Short chain fatty acids were measured in mouse intestinal samples at section. Luminal content of the caecum (ten mice per group) was collected in H_3_PO_4_ and isocaproic acid (as an internal standard) containing buffer solution. Samples were stored at -20°C until further processing. The day of analysis, samples were thawed, centrifuged at 14.000 rpm (5 min), and supernatant was collected and stored at 5°C. The samples were then subjected to gas chromatography (Fisons HRGC Mega 2, CE Instruments, Milan, Italy) at 190°C using a glass column fitted with Chromosorb 101 with a carrier gas (N_2_ saturated with methanoic acid).

### Microbial community composition

Metagenomic DNA was extracted from the ileum, caecum and colon samples (4 mice per diet) using the repeated bead beating plus column (RBB+C) method [18]. The amount that was used for DNA extraction for the ileum was all what we could squeeze out, the caecum roughly a quarter of ceacal content (0.1–0.2 grams) and for the colon a colonic pellet (0.1 grams). The microbial communities in the intestinal samples were analysed with the Mouse Intestinal Tract Chip (MITChip). This phylogenetic microarray consists of 3,580 different oligonucleotides specific for the mouse intestinal microbiota ([19]. The array targets the V1 to V6 region of 16S rRNA genes of bacteria. The 16S rRNA genes were amplified from 20 ng of intestinal metagenomic DNA with the primers *T7prom*-Bact-27-F and Uni-1492-R (Table 2). The PCR products obtained were transcribed, labelled with Cy3 and Cy5 dyes and fragmented. Finally, the samples were hybridized on the arrays at 62.5°C for 16 hours in a rotation oven (Agilent Technologies, Amstelveen, the Netherlands). After the slides were washed and scanned, data was extracted with the Agilent Feature Extraction software (version 9.1). The data was normalized and analysed using a set of R-based scripts combined with a custom-designed relational database, which operates under the MySQL database management system.

**Table 2:** List of primers used in this study [20–22].

Primer name	Sequence	Application
T7prom-Bact-27-F	5’-TGA ATT GTA ATA CGA CTC ACT ATA GGG GTT TGA TCC TGG CTC AG–3’	MITChip
Uni-1492-R	5’-CGG CTA CCT TGT TAC GAC-3’	MITChip
PROK1492R	5' -GGW TAC CTT GTT ACG ACT T-3'	QPCR
BACT1369F	5'-CGG TGA ATA CGT TCY CGG-3'	QPCR

### RNA extraction, mRNA enrichment, cDNA synthesis and illumina sequencing

Four intestinal caecum content samples from each dietary treatment were used to analyze the metatranscriptome activity profiles. The RNA was extracted from 0.1–0.2 grams of ceacal content. The content was suspended in 500 μL ice-cold TE buffer (Tris-HCL pH 7.6, EDTA pH 8.0). Total RNA was obtained via the Macaloid-based RNA isolation protocol [23,24] with in addition the use of Phase Lock Gel heavy tubes (5 Prime GmbH, Germany) during phase separation. The RNA purification was performed using the RNAeasy mini kit (Qiagen, USA), including an on-column DNAseI (Roche, Germany) treatment [23]. The total RNA was eluted in 30 μL ice-cold TE buffer and the RNA quantity and quality were assessed using a NanoDrop ND-1000 spectrophotometer (Nanodrop Technologies, Wilmington, USA) and Experion RNA Stdsens analysis kit (Biorad Laboratories Inc., USA), respectively. The mRNA enrichment was performed using the mRNA enrichment kit (MICROBExpressTM, Ambion, Applied Biosystem, the Netherlands) according to the manufacturer’s protocol. Following the enrichment, the quantity and quality of the RNA were assessed again (see above) to confirm the efficacy of the mRNA enrichment procedure. One μg of the enriched mRNA sample was used to reverse-transcribe the RNA to cDNA, and subsequently generate double stranded cDNA using the SuperScript® Double-Stranded cDNA Synthesis kit (Invitrogen, the Netherlands, 11917–010), and employing the SuperScript® III Reverse Transcriptase (Invitrogen, the Netherlands 18080–044) and random priming using random hexamers (Invitrogen, 48190–011) [24–26]. To remove RNA from the double stranded cDNA preparations, RNAse A (Roche, Germany) treatment was performed, followed by phenol-chloroform extraction and subsequent cDNA purification and concentration by ethanol precipitation. The product was checked on 1% agarose gel and 3 to 8 μg of cDNA was sent for sequencing (GATC Biotech, Germany). Single read Illumina Libraries were prepared from the double-stranded cDNA according to the ChiP-seq protocol [27] with insert sizes between 200–300 bp, suing barcoded tags for library constructions to enable parallel sequencing (GATC Biotech, Germany). Sequencing was performed using Illumina Hiseq2000 and using 5 pM concentration of the library and the single-end protocol [24].

### Data filtering

Sequencing generated between 13.4 and 177 million reads per sample. The data set supporting the results of this article is available in the NCBI small reads archive (sra) repository, under accession number SRP043409. The data was filtered, assembled, annotated and classified as previously described (Davids *et al*., 2016). Briefly, the taxonomic origin of the contigs was assigned by alignment of the predicted protein sequences with the NCBI’s non-redundant database, retrieving the taxonomic family classification of the protein sequence with the highest similarity. Classification up to family level was chosen, determined in our previous study to be able to have enough confidence in the assignment and still enough precision in the involved community member [28]. Functional annotation was achieved by assignment of KEGG orthology identifiers to all predicted protein sequences, using the KEGG KAAS server (Moriya *et al*., 2007; http://www.genome.jp/tools/kaas). Expression levels of individual genes were determined by aligning the mRNA reads with the assembled protein-encoding contigs and enumerating the total amount of nucleotides aligned with the corresponding ORFs.

### Functional analysis and metabolic pathway mapping

The expression levels of each of the protein encoding regions in each of the samples were normalized by scaling each gene by the total number of nucleotides that were mapped to ORFs of that same sample. The normalized expression levels were used to determine both relative activity and functional analysis in Canoco 5.0. Metabolic mapping of the metatranscriptome profiles was performed quantitatively by mapping the KEGG annotated protein sequences onto metabolic pathway maps using the iPath v2 module (http://pathways.embl.de/iPath2.cgi#). Gene expression levels of the metabolic pathways was indicated by the line width, which was determined from the log 10 values of the read count per KEGG annotated protein.

Specific functions were selected on KEGG annotations and relative activity was mapped in bar plots or boxplots, statistical significance was tested using the Wilcoxon test.

## Results & discussion

Long-term effects of high protein diets on the composition and activity of the gut microbiota were assessed in a 12-week dietary intervention study in mice. The effects of high protein (casein) were studied both in a low- and high-fat dietary background, to evaluate whether dietary fat content affects the outcome of the high protein intervention. Male C57BL/6J mice of 10 weeks (young adults) were given the control, a normal protein and low fat diet, for two weeks, followed by a 12 week dietary intervention. Mice were divided into four groups (n = 10 per group), receiving the control diet (normal protein low fat, NPLF), a normal protein high fat diet (NPHF), a high protein low fat diet (HPLF) or a high protein high fat diet (HPHF) (Table 1). The effects of these dietary interventions were measured in terms of microbiota composition and activity, the latter being determined based on the fermentation output, measured by luminal SCFA levels, as well as transcription-activity levels.

### Dietary proteins differentially modulate luminal SCFA levels

The main fermentation metabolites of dietary protein are short chain fatty acids, predominated by acetate, propionate and butyrate. During protein fermentation also BCFA are formed from the degradation of branched amino acids. Gas chromatography was used to measure concentrations of acetate, propionate, butyrate, valerate and the BCFA iso-butyrate and iso-valerate in caecal luminal content of mice receiving the different diets.

**Fig 1:**
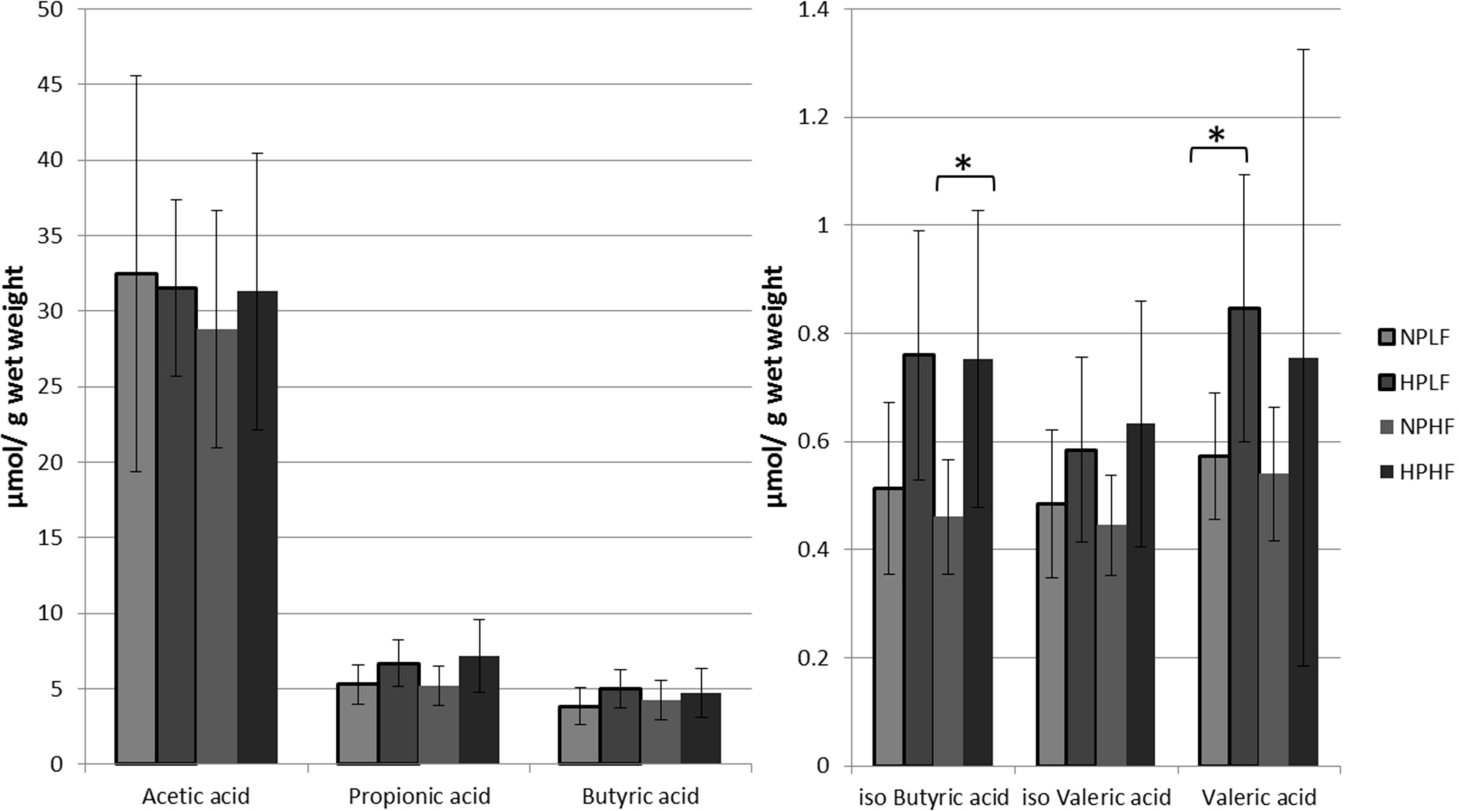
Caecal luminal SCFA concentrations in μmol/g content measured with gas chromatography. * Indicates significance between two groups (Ttest, P < 0.05). In light grey are both the normal protein diets: Normal Protein-Low Fat (NPLF) and Normal Protein-High Fat (NPHF). In dark grey are both the high protein diets: (High Protein-Low Fat (HPLF) and High Protein-High Fat (HPHF).

Overall, the high protein diets led to an increase of SCFA and BCFA concentrations in the caecum (Fig 1). However, due to the high variation between individual mice, a significant increase could only be detected for iso-butyrate in the HPHF group relative to the NPHF group, and for valeric acid in the HPLF group compared to the NPLF group. In these analyses it should also be taken into account that the relative amount of cornstarch in the diets was drastically decreased in diets with increased protein and fat content. Notably, this implies that despite the reduced availability of cornstarch for microbial fermentation in the HPHF diet, the microbiota still generated higher overall SCFA and BCFA concentrations that likely derive from protein fermentation. Protein fermentation has been proposed to account for up to 17% of the overall SCFA production in the caecum (Macfarlane, *et al*., 1992). Apparently, high level protein fermentation by the microbiota supports higher BCFA concentrations in the caecal lumen as compared to fermentation of the alternative, readily digestible nutrients (e.g. cornstarch).

### Dietary proteins modulate intestinal microbiota composition

Intestinal content of four mice per dietary treatment was used to analyze the microbiota composition after 12 weeks of diet intervention, separately analyzing ileal, caecal and colonic microbiota, using the MITChip platform. This platform employs a 16S rRNA targeted phylogenetic microarray designed for the comprehensive and deep profiling of mouse intestinal microbiota composition [29,30,20,31]. MITChip analysis revealed clearly distinct microbiota composition profiles in the ileum as compared to those obtained for caecal and colonic content. Notably, the ileal microbiota composition patterns did not cluster according to the dietary intervention, whereas the caecal and colonic microbiota profiles tended to cluster closely together and sub-clustered according to the diet (S1 Fig). The caecum is considered as the intestinal region where most prominent microbiota fermentation takes place (Nguyen *et al*., 2015, Hugenholtz *et al*., 2016). Moreover, the caecum allowed the extraction of an amount of intestinal content that provided RNA amounts compatible with metatranscriptome analysis. Therefore, our analyses focused on the composition and metatranscriptome analysis of the microbiota residing in this intestinal region (see below).

**Fig 2:**
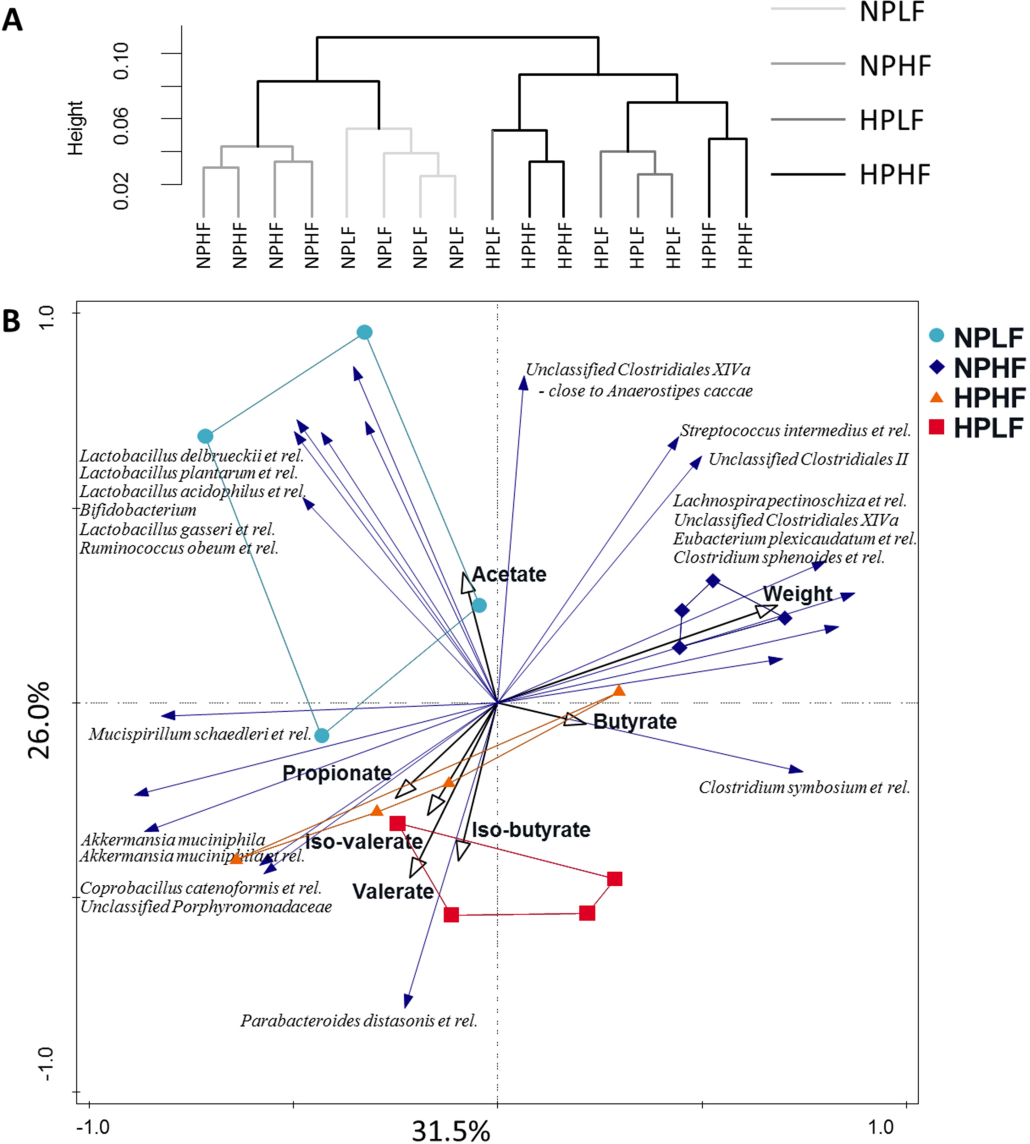
2a. Hierarchical clustering of the caecal samples on log10 transformed probe level data of the MITChip. The clustering was made using Pearson distance and via the Ward linking method. **2b.** Redundancy analysis on genus-level phylogenetic groups of the MITChip data. The explanatory variables were the dietary groups, weight of the mice, acetate, propionate, butyrate, valerate, iso-butyrate and iso-valerate. Explanatory variables account for 85.8% of the total variation, and in the figure 57.5% of the variation is shown. The samples are colour coded per dietary group: in light blue NPLF, dark blue NPHF, in orange HPHF and in red HPLF. The nominal variables are indicated in black arrows and the species that had 50% or more of their variation on the first two axes are shown with blue arrows.

MITChip analyses revealed distinct microbiota composition at probe level in the caecum from animals that were fed the normal protein (NPLF and NPHF) or high protein content (HPLF and HPHF) diets (Fig 2A). Notably, within the NP diets the fat level resulted in distinct clustering of the microbiota from mice on the low (NPLF) and high (NPHF) fat content diets, whereas microbiota composition at probe level failed to discriminate the HP diets on basis of their fat-content. This finding illustrates that within the NP diet context the other main nutritional component (i.e., fat content) has a prominent influence on the microbiota, whereas this effect is lost or overruled by the high protein content in the HP diet context.

### Microbiota and fermentation-metabolite data integration

In order to relate changes in caecal microbiota composition to the different diets, the weight of the mice (previously published in [17]), as well as to SCFA and BCFA as the main metabolites of microbial fermentation, hybridization signals of in total 96 genus-level phylogenetic groups were subjected to redundancy analysis (RDA). The RDA included the concentrations of acetate, propionate, butyrate, iso-butyrate, valerate, iso-valerate, the weight of the mice and the diets as explanatory variables. Overall, these explanatory variables accounted for 85.8% of the total variation, 57.5% of which was covered by the first two canonical axes (Fig 2B). Samples from animals consuming different diets clustered separately along these canonical axes, and the LFNP, HFNP and HFHP diets as explanatory variables had a significant (Monte Carlo Permutation test, p<0.05) impact on the total variation of the data. Microbial groups that correlated with the LFNP diet included *Bifidobacterium*, *Lactobacillus delbrueckii* et rel., *Lactobacillus plantarum* et rel., *Lactobacillus acidophilus* et rel., *Lactobacillus gasseri* et rel., and *Ruminococcus obeum* et rel. Notably, these groups also correlated with caecal acetate concentrations. Notably, *Ruminococcus obeum* can use a wide range of sugars [32], and lactobacilli are known for their rapid sugar import and metabolism [33]. The higher abundances of these typical saccharolytic bacterial groups suggest that in the mice on the LFNP diet, the higher relative amounts of the carbohydrates in this diet, i.e., cornstarch and sucrose, are incompletely digested and absorbed in the small intestine, and thus available for microbial fermentation in the caecum. The HFNP diet as well as the body-weight of the mice strongly correlated with higher abundances of several groups within *Clostridium* cluster XIVa. Moreover these groups anti-correlate with the HP dietary groups, similarly seen in rats on high-protein diets [13,15]. Inversely, *Akkermansia muciniphila* was anti-correlated with the HFNP diet and body-weight, where *A*. *muciniphila* was reported before to decrease in abundance in diet induced obese mice [34,29]. Samples taken from animals fed the HP diets grouped closely together and correlated with elevated levels of BCFA as well as with a higher abundance of *Parabacteroides distasonis* et rel. Both *Parabacteroides distasonis* and the Unclassified *Porphyromonadaceae* belong to *Porphyromonadaceae*, which is decreasing in abundance in the mice fed NPHF (see metatranscriptome section below).

### Effect of high protein and high fat on overall microbial metatrancriptome patterns

The activity profiles of the microbiota obtained from the caecum of mice were determined by metatranscriptome analysis in each of the diet-groups at the end of the dietary intervention period. To this end, the caecal contents of four mice of the LFHP, HFHP, HFNP and three mice of the LFNP group were used for RNA extraction, mRNA enrichment, cDNA synthesis and illumina metatranscriptome sequencing. The sequencing efforts generated between 3.4 x 10^5^ and 19.2 x 10^5^ (with a single outlier of 5.6 x 10^6^) high quality mRNA-derived sequence reads per sample. To determine the function and taxonomy of these reads, they were merged and *de novo* assembled into larger contigs using the pipeline described previously [28], creating a single contig reference set for all samples. A total of approximately 3.8 x 10^4^ contigs could be assembled with an overall length of 29.8 x 10^6^ bases (n50 = 945). These contigs encoded a total of approximately 5.5 x 10^4^ predicted protein-encoding open reading frames. Between 54% and 74% of the mRNA reads could be assigned to the predicted protein-encoding genes (Table 3). Clustering of the expression levels of the different samples resembled that of the MITChip-derived microbiota composition profiles (S2 Fig). Therefore, analogous to microbial composition, the microbiota activity profiling enabled the detection of the impact of the dietary fat content in the NP diets, whereas this effect of the fat content appeared to be lost or overruled by the high protein content in the HP diets.

**Table 3:** Number of reads from the illumina sequencing, rRNA filtering, assembly of the mRNA reads and functional assignment of the coded proteins on the assembled mRNA contigs.

Sample name	Total reads	mRNA	Assembled mRNA reads	Bacterial protein coding in assembled contigs
HPLF_3	3.49E+07	7.14E+05	73.7%	83.1%
NPLF_2	3.29E+07	1.58E+06	66.2%	83.1%
NPLF_3	3.11E+07	1.92E+06	64.0%	88.6%
HPLF_4	1.93E+07	3.83E+05	64.7%	80.2%
HPLF_1	1.81E+07	4.02E+05	76.4%	78.2%
NPHF_1	1.40E+07	4.48E+05	62.1%	83.7%
NPLF_1	1.61E+07	7.28E+05	62.6%	80.1%
HPHF_1	2.49E+07	6.38E+05	73.2%	73.0%
HPHF_2	1.77E+08	5.58E+06	73.3%	82.0%
HPHF_3	1.34E+07	3.37E+05	75.8%	75.5%
NPHF_2	1.83E+07	4.03E+05	68.0%	74.9%
NPHF_3	2.35E+07	5.60E+05	72.2%	59.7%
HPLF_2	3.29E+07	6.78E+05	75.0%	70.6%
NPHF_4	2.40E+07	5.99E+05	64.7%	65.2%
HPHF_4	1.77E+07	5.78E+05	80.5%	53.1%

### Effect of high protein and high fat diets on microbiota function profiles

To focus only on functions that are differentially expressed within the microbiota as a function of the different diets, we employed an in house R script to detect KEGG modules that are differentially expressed. Remarkably, the different fat levels in the diets (HF versus LF) were not significantly correlated to any differentially expressed KEGG modules. In contrast, the comparison of samples derived from NP and HP diet fed mice enabled the detection of KEGG modules that displayed significantly different expression levels. The KEGG modules enriched in the NP diet derived samples were all associated with sugar metabolism, whereas the modules enriched in the HP diet derived samples were consistently associated with protein metabolism (Table 4). These findings are in good agreement with the clustering analyses as well as the anticipated dietary impacts on the nutrients available for fermentation in the caecum of the mice that were fed on the different diets.

**Table 4:** Enriched modules within the NP or HP dataset. Significant higher KEGG numbers (Ttest, P<0.05) were taken together for the NP diets or HP diets and the likelihood for the expression of a module was calculated (with P<0.05).

	Module	nr of KEGGs in modules	nr of KEGGs found	Module explanation
**Enriched in NP**	M00377	10	4	Reductive acetyl-CoA pathway (Wood-Ljungdahl pathway) [PATH:map01200 map00720]
	M00422	5	3	Acetyl-CoA pathway, CO2 = > acetyl-CoA [PATH:map00680]
	M00196	4	3	Multiple sugar transport system [PATH:map02010] [BR:ko02000]
**Enriched in HP**	M00018	10	4	Threonine biosynthesis, aspartate = > homoserine = > threonine [PATH:map01230 map00260]
	M00299	4	3	Spermidine/putrescine transport system [PATH:map02010] [BR:ko02000]
	M00236	3	3	Putative polar amino acid transport system [BR:ko02000]

### Effect of high protein and high fat on microbial community activity

The microbial families *Lachnospiraceae*, *Erysipelotrichaceae* and *Clostridiaceae* dominated in the overall activity associated with the degradation of proteins, supporting their high relative contribution in the total mRNA activity profile in all the samples (S3A Fig). *Erysipelotrichaceae* has relatively higher abundances in the HP diets, while the abundances of *Lachnospiraceae* decreased in the HP diets. Many of the butyrate producing bacteria belong to *Lachnospiraceae*, *Ruminococcaceae* and *Eubacteriaceae* and all of these families tended to decrease in the HP, supporting earlier studies in rodents [13,15,14].

**Fig 3:**
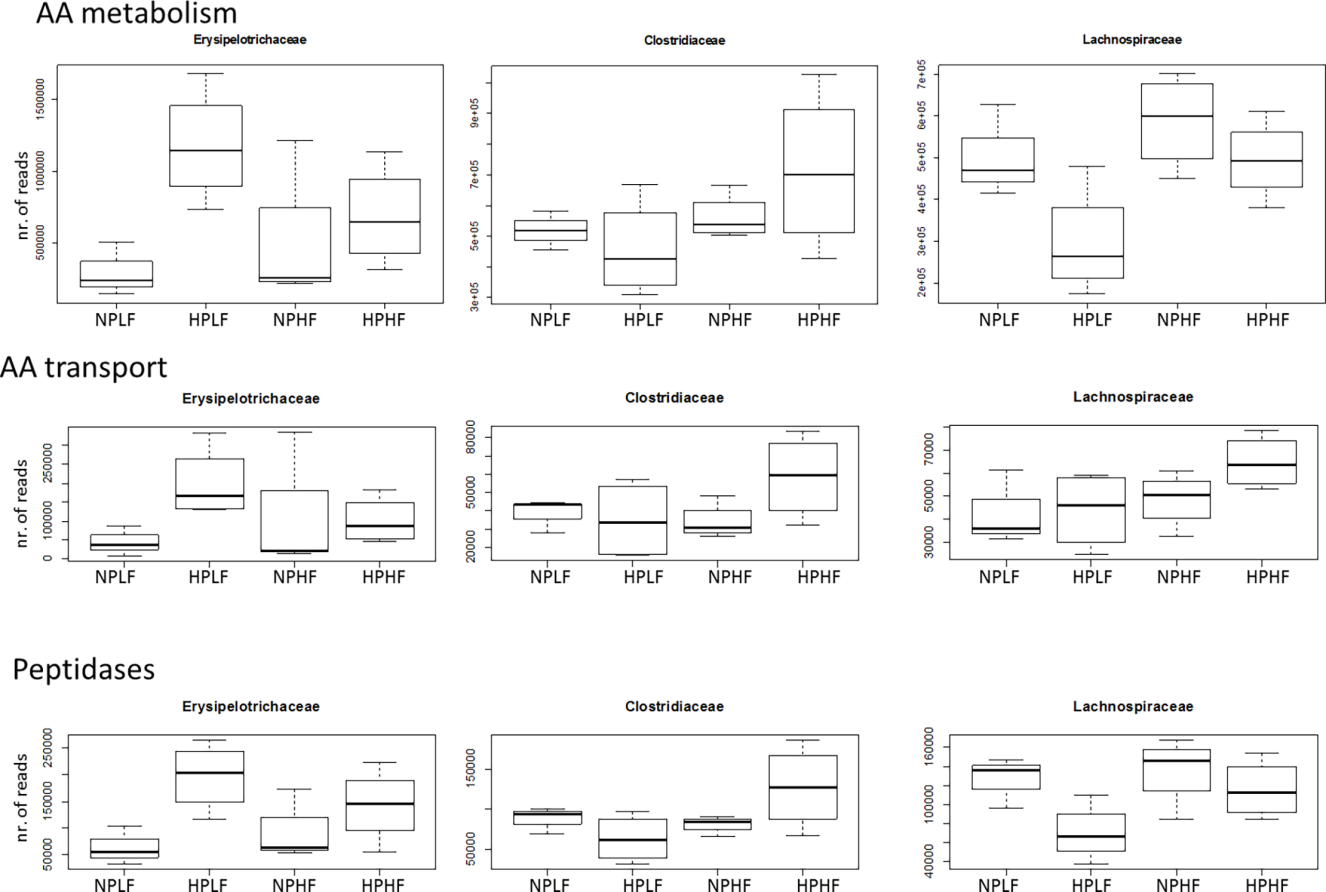
Expression levels of peptidases, amino acid metabolism related proteins and amino acid transporters originating from the *Erysipelotrichaceae*, *Clostridiaceae* and *Lachnospiraceae*. All the genes that were predicted according to their KEGG orthology to belong to either peptidases, amino acid metabolism or amino acid transporters, were accumulated and plotted in a box plot. On the vertical axis the number of reads are depicted.

To further support an eventual role of microbial groups in protein fermentation, the metatranscriptome datasets were mined for genes involved in proteolysis (S3B Fig), amino acid metabolism (S3C Fig), and amino acid transport (S3D Fig), based on their KEGG orthology annotation. Especially the *Erysipelotrichaceae* displayed an increased contribution to the overall protein degradation, which was most prominently detected in samples obtained from mice that were fed the HPLF diet, whereas a similar trend was observed in the samples obtained from HPHF-fed mice (Fig 3). Conversely, the *Lachnospiraceae* tended to decrease their relative contribution to the overall protein degradation, illustrated by the reduced expression of peptidase and amino acid metabolism functions by this bacterial group in the HPLF compared to the NPLF diet. These two families display opposite features in response to the protein level in the diet, suggesting amino acid catabolism activity in *Erysipelotrichaceae* and carbohydrate dependence in *Lachnosipraceae*. Remarkably *Bacteroidetes* families hardly contributed to the overall protein catabolism (S3B, S3C and S3D Fig), even though their abundance was correlated with the HP diets in the MITChip analysis. Although members of *Bacteroides* have previously been associated with amino acid intake in humans [11] and increased in mice on a high-protein diet [14], in the present study this microbial group did not seem to prominently participate in transport and catabolism of amino acids.

In contrast to the observed increased relative abundance of protein degradation related transcripts, one of the glycolysis-associated genes encoding the 6-phosphofructokinase, was less expressed in the HPLF diet, which was mainly due to the decreased expression observed for *Lachnospiraceae* (S3E Fig). The *Lachnospiraceae* appeared to be less effective in the utilization of protein sources and were more dependent on carbohydrate utilization in the gut. These results are in agreement with the observation that the *Clostridium coccoides* group, belonging to the *Lachnospiraceae*, was found in reduced abundance in response to high protein levels in the diet in rat experiments [13].

To investigate the specific activity patterns of the three predominantly active microbial families, i.e. the *Lachnospiraceae*, *Erysipelotrichaceae* and *Clostridiaceae*, in more detail, their specific-activity was plotted on the metabolic map available in the iPATH software suite (S4 Fig). Nevertheless, each of the microbial families displayed quite distinct expression patterns. For example, *Lachnospiraceae* strongly expressed genes coding for enzymes involved in the conversion of phosphoenolpyruvate to oxaloacetate, and lipid biosynthesis activity, whereas the *Erysipelotrichaceae* hardly displayed these activities. In turn, the *Erysipelotrichaceae* appeared to be much more focused on the conversion of malate, fumarate and succinate. Finally, the predominant *Clostridiaceae* representatives expressed both these activities, as well as a broad spectrum of pathways related to amino acid metabolism. Notably, each of the iPATH mapped pathways for the activity patterns of the families *Lachnospiraceae*, *Erysipelotrichaceae* and *Clostridiaceae* appeared to display gaps, suggesting incomplete or incorrect annotations of genes in the metatranscriptome data. This may either be due to their low *in situ* expression levels or incorrect functional annotation, or taxonomic misclassification of the predicted proteins. Many of the inaccuracies in assignment of function and taxonomic origin of a sequence derived from the mouse cecum is likely due to inaccurate annotation assignments in the NCBI database as well as the substantial dissimilarity between the reference genomes and the metatranscriptome data obtained from the microbial community residing in the murine intestinal tract. Additionally, the limited depth of metatranscriptome analysis could also provide a limitation in the detection of complete and/or lowly expressed pathways.

Overall, members of the *Erysipelotrichaceae* appeared to benefit most significantly from the HPLF diet, and the expression profiles assigned to this group confirmed its focus on pathways for the degradation of a several amino acids. In the HPHF diet group, possibly due to the higher fat content, the advantage of the *Erysipelotrichaceae* appeared to be reduced relative to the LF diets and they appeared to be at least partially replaced by members of the *Clostridiaceae* that expressed a wider range of amino acid catabolic pathways.

**Fig 4:**
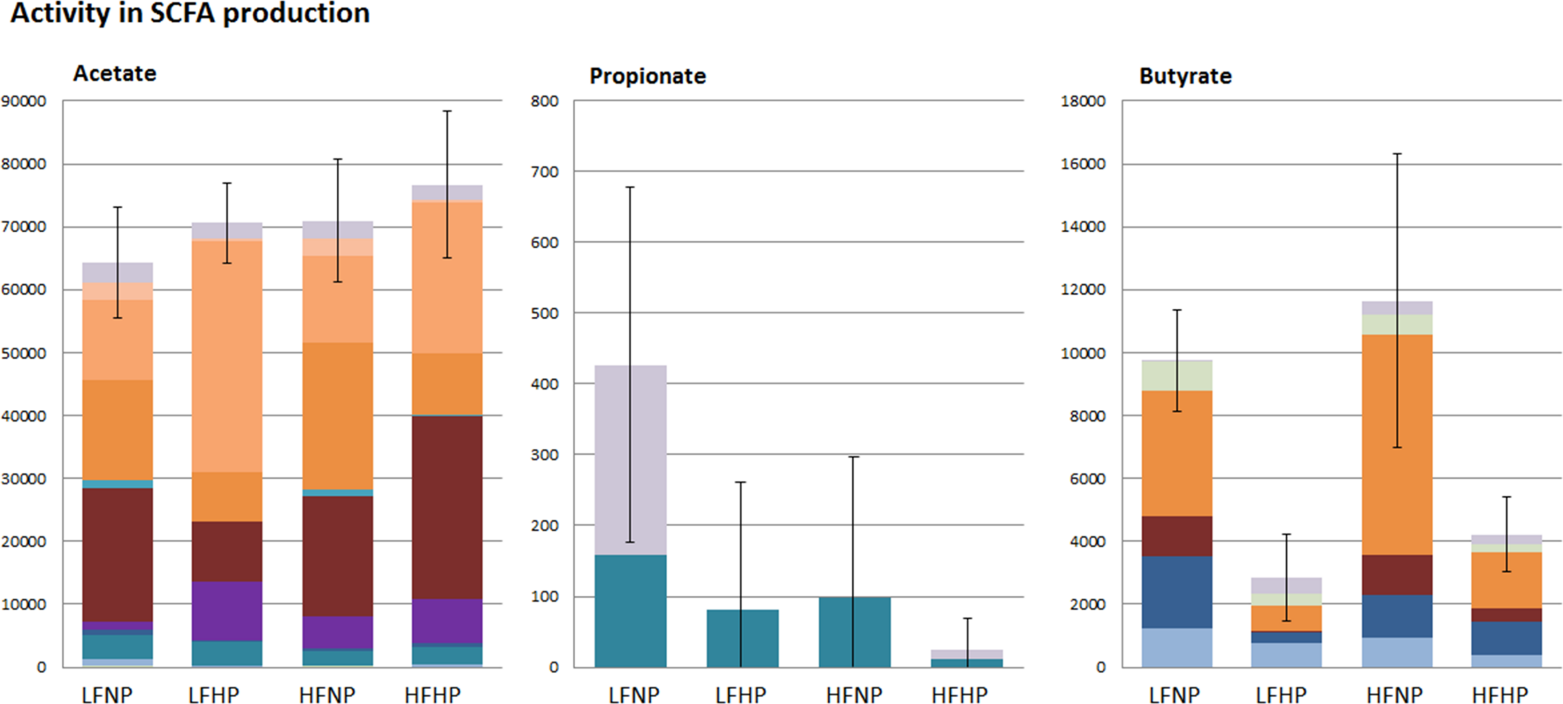
Average relative abundances of metatranscriptome derived families that have activity towards SCFA production. Acetate production via acetate kinase, acetyl-CoA synthase and phosphate acetyltransferase (K00925, K00625, K01895, K13788; Propionate production via propionate CoA-transferase and acetyl-CoA synthase (K01895, K01026); Butyrate production via butyrate kinase, acetoacetate CoA-transferase and phosphate butyryltransferase (K01034, K01035, K01896, K00929, K00634). Error bars are SD of total activity per type of SCFA (Acetate, Propionate or Butyrate) in each dietary group.

The observed expression profiles of genes encoding enzymes involved in SCFA production suggest that *Erysipelotrichaceae* produced predominantly acetate as the main end product of protein catabolism, whereas *Clostridiaceae* and *Lachnospiraceae* were predicted to produce both acetate and butyrate (Fig 4). However, the *Erysipelotrichaceae* expressed genes involved acetyl-coA to butyryl-coA conversion, using the energy-conserving crotonyl-coA pathway [35], suggesting that also this family contributes to butyrate production. Notably, the expression of genes encoding butyrate-kinase and butyryl-CoA:acetate-CoA-transferase that are also involved in the crotonyl-CoA pathway was not detected in *Erysipelotrichaceae*, which is likely due to the erroneous annotations of the homologous acetate-kinase and other SCFA transferase functions in this bacterial group [36–38]. Genes that encode the enzymes required for propionate production appeared barely expressed, and were exclusively assigned to the bacterial families *Porphyromonadaceae* and *Sphingomonadaceae*. The very low expression detected for the propionate production pathway may indicate that for a more complete reconstruction of the microbiome activity profiles, metatranscriptome datasets with a higher depth of analysis would be required. Analogously, we failed to detect the expression of genes encoding enzymes involved in BCFA production, which may also require a higher depth of metatranscriptome analysis considering that concentrations of these metabolites were 2–3 orders of magnitude lower than those of acetate, propionate and butyrate (Fig 1). Alternatively, some of the enzymes involved might not be classified accurately in the KEGG system and may therefore be missed. Despite these limitations in assigning specific enzyme and pathways, the enriched modules associated with amino acid metabolism in the metatranscriptome data established the role of the microbiota in the fermentation of dietary proteins. The unaltered pattern of specific activities assigned to *Lachnospiraceae*, *Erysipelotrichaceae* and *Clostridiaceae* indicates their consistent contribution to the *in situ* protein catabolism despite the substantial differences in protein content of the respective diets.

## Conclusion

Here we show that prolonged feeding of high protein level diets for a period of 12 weeks exerted a prominent effect on the composition of caecal microbiota and its protein fermentation capacity, supporting elevated SCFA production in the caecal lumen as compared to fermentation of the alternative nutrients (i.e., cornstarch or fat). In addition, the data also revealed a prominent influence on the microbiota composition of the fat content in diets that contain normal protein levels. The microbial community members most active in the different diet groups belonged to the families of the *Lachnospiraceae*, *Erysipelotrichaceae* and *Clostridiaceae*, and were predicted to produce mainly acetate and butyrate based on the metatranscriptome profiles. The relative activity of especially the *Erysipelotrichaceae* appeared to be increased in mice consuming the high protein diets, although the *Clostridiaceae* expressed a wider range of different amino acid metabolism associated pathways. Moreover the total activity of the genes involved in these metabolites does not correspond to the concentration of acetate, propionate and butyrate measured in the caecum. So there is need for metabolite flux measurements, rather than luminal steady state concentrations. Furthermore a more complete reconstruction of the microbiome activity profiles is necessary to provide a comprehensive understanding of the role of *Erysipelotrichaceae* and *Clostridiaceae* in the *in situ* fermentation of dietary protein. Such improved understanding would be strongly facilitated by pure and mixed *in vitro* culture studies using representatives of these microbial families to better characterize their metabolic repertoire and the genes involved in the relevant pathways, which would enable a more accurate metatranscriptome mapping. In conclusion, the data presented here provide clear metabolic indications concerning the microbial groups involved in protein catabolism in the intestine, but more complete understanding of the precise role of these microbes will require a better understanding of their physiological characteristics and may also require metatranscriptome datasets with a higher depth of analysis.

## Supporting information

S1 Fig Hierarchical clustering of all the samples on log10 transformed probe level data of the MITChip. The clustering was made using pearson similarity and via the Ward linking method. The letters below or indicative for the origin of the sample: C for caecum, L for colon and I for ileum.
(TIF)

S2 Fig Hierarchical clustering on normalized (annotated) bacterial metatranscriptome data. Clustering of the 15 samples was done using pearson similarity and via the Ward linking method. In light grey are both the normal protein diets: Normal Protein-Low Fat (NPLF) and Normal Protein-High Fat (NPHF). In dark gray are both the high protein diets: (High Protein-Low Fat (HPLF) and High Protein-High Fat (HPHF).
(TIF)

S3 Fig **S3a** Relative abundance of total metatranscriptome (activity) on family level. All the genes that were predicted with a KEGG orthology were accumulated and their taxonomic origin on family level is plotted here. Families with activity over 0.5% abundances in any of the conditions are plotted. **S3bcd.** Relative abundance of families expressing peptidases (b), amino acid metabolism related proteins (c) and amino acid transporters (d). All the genes that were predicted according to their KEGG orthology to belong to either peptidases, amino acid metabolism or amino acid transporters, were accumulated and their taxonomic origin on family level is plotted here. Families with activity over 0.5% abundances in any of the conditions are plotted. **S3e.** Expression level of 6-phosphofructokinase in the glycolysis pathway. This enzyme catalizes a step in the glycolytic pathway and its gene was transcribed at a lower level in the HPLF diet, which was mainly due to the decreased expression from *Lachnospiraceae*.
(TIF)

S4 Fig Metabolic activity of *Lachnospiraceae*: Green; *Clostridiaceae*: Red; *Erysipelotrichaceae*: Blue. The expression patterns of all the KEGG numbers per individual family were plotted in with the iPATH software suite. In each of the microbial families unique expression patterns were found, which are indicated within the grey circles. *Lachnospiraceae* strongly expressed genes coding for enzymes involved in the conversion of phosphoenolpyruvate to oxaloacetate, and lipid biosynthesis activity. The *Erysipelotrichaceae* appeared to be much more focused on the conversion of malate, fumarate and succinate. The *Clostridiaceae* representatives were concluded to express both these features and a broad spectrum of pathways related to amino acid metabolism.
(TIF)
