# Supplementary figures and images for: Metatranscriptome analysis of the microbial fermentation of dietary milk proteins in the murine gut
Source: PLoS One. 2018 Apr 17;13(4):e0194066. doi: 10.1371/journal.pone.0194066 (PMC5903625; doi:10.1371/journal.pone.0194066)

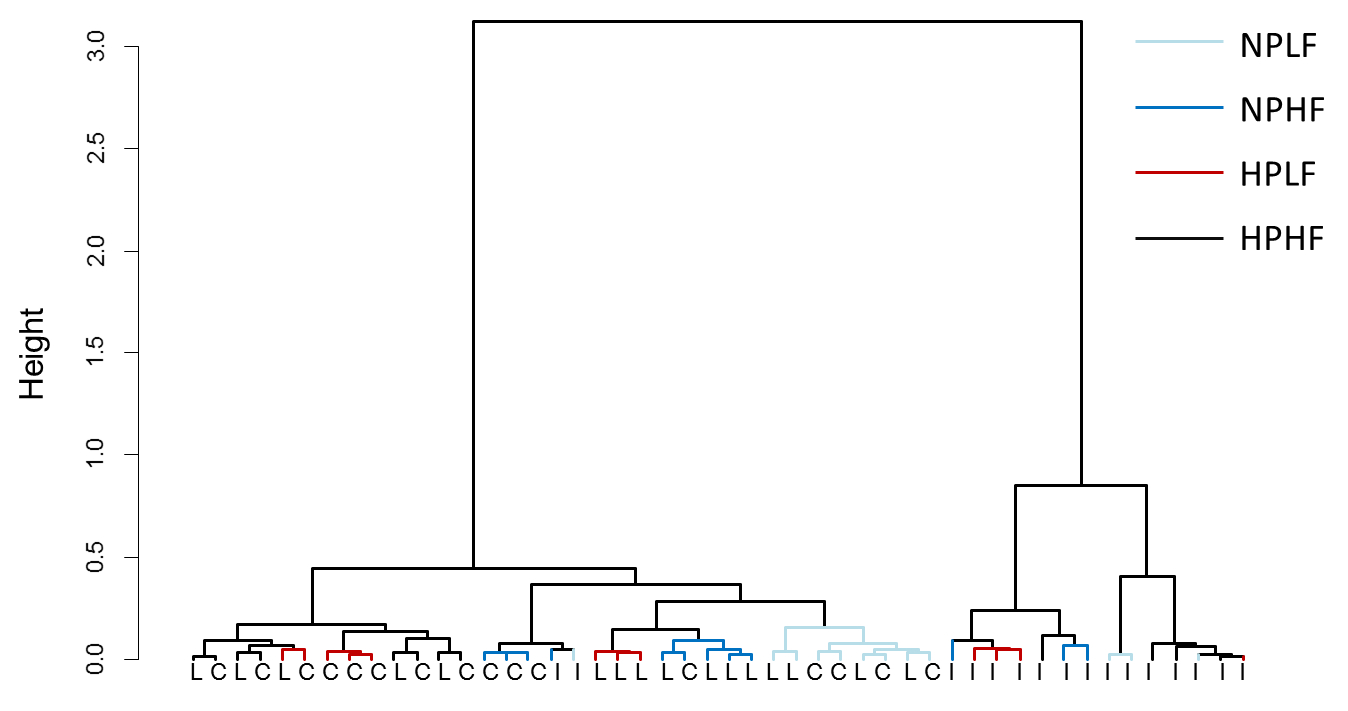

Supplement: S1 Fig — The clustering was made using pearson similarity and via the Ward linking method. The letters below or indicative for the origin of the sample: C for caecum, L for colon and I for ileum. (TIF) [file pone.0194066.s001.tif]

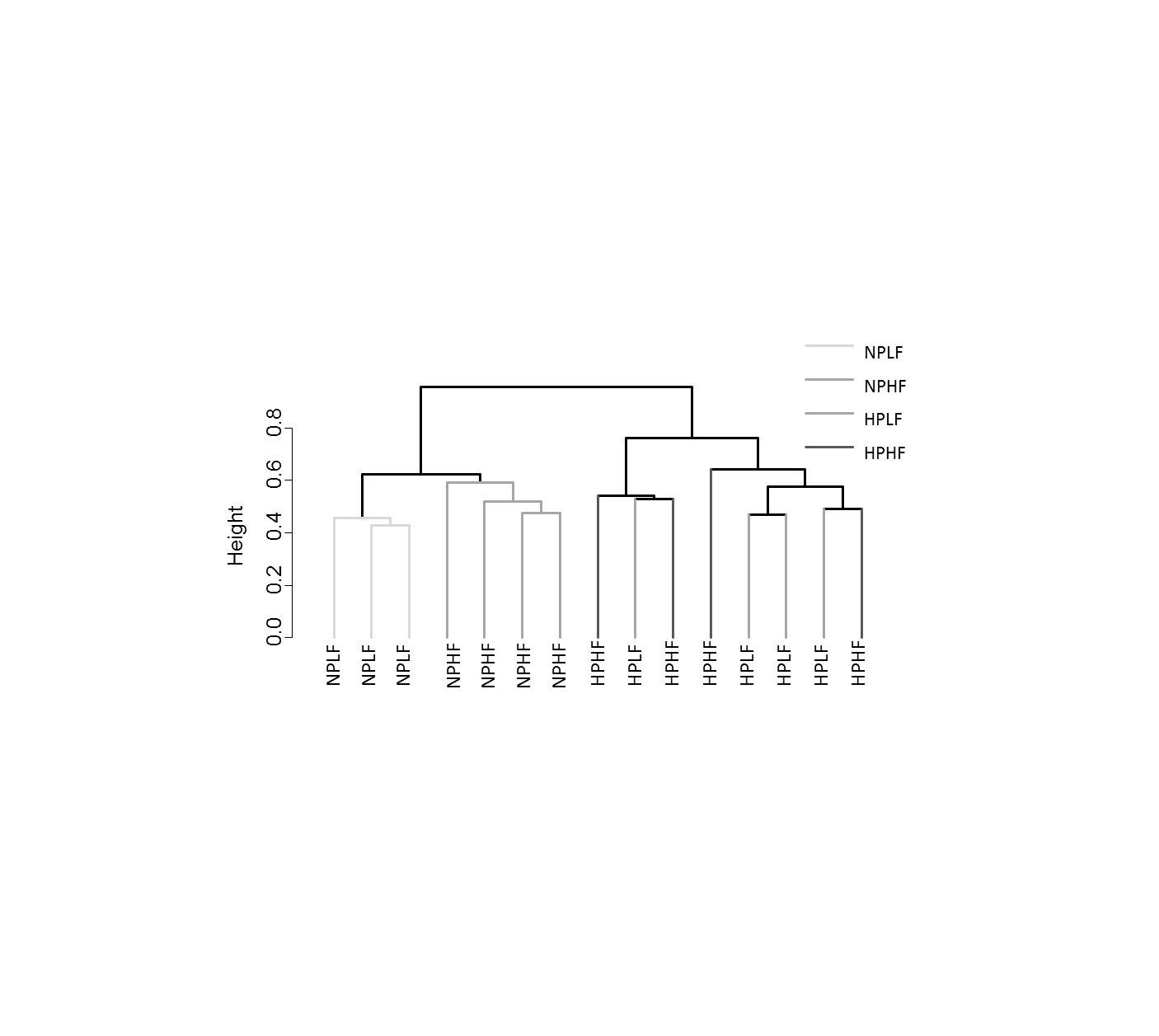

Supplement: S2 Fig — Clustering of the 15 samples was done using pearson similarity and via the Ward linking method. In light grey are both the normal protein diets: Normal Protein-Low Fat (NPLF) and Normal Protein-High Fat (NPHF). In dark gray are both the high protein diets: (High Protein-Low Fat (HPLF) and High Protein-High Fat (HPHF). (TIF) [file pone.0194066.s002.tif]

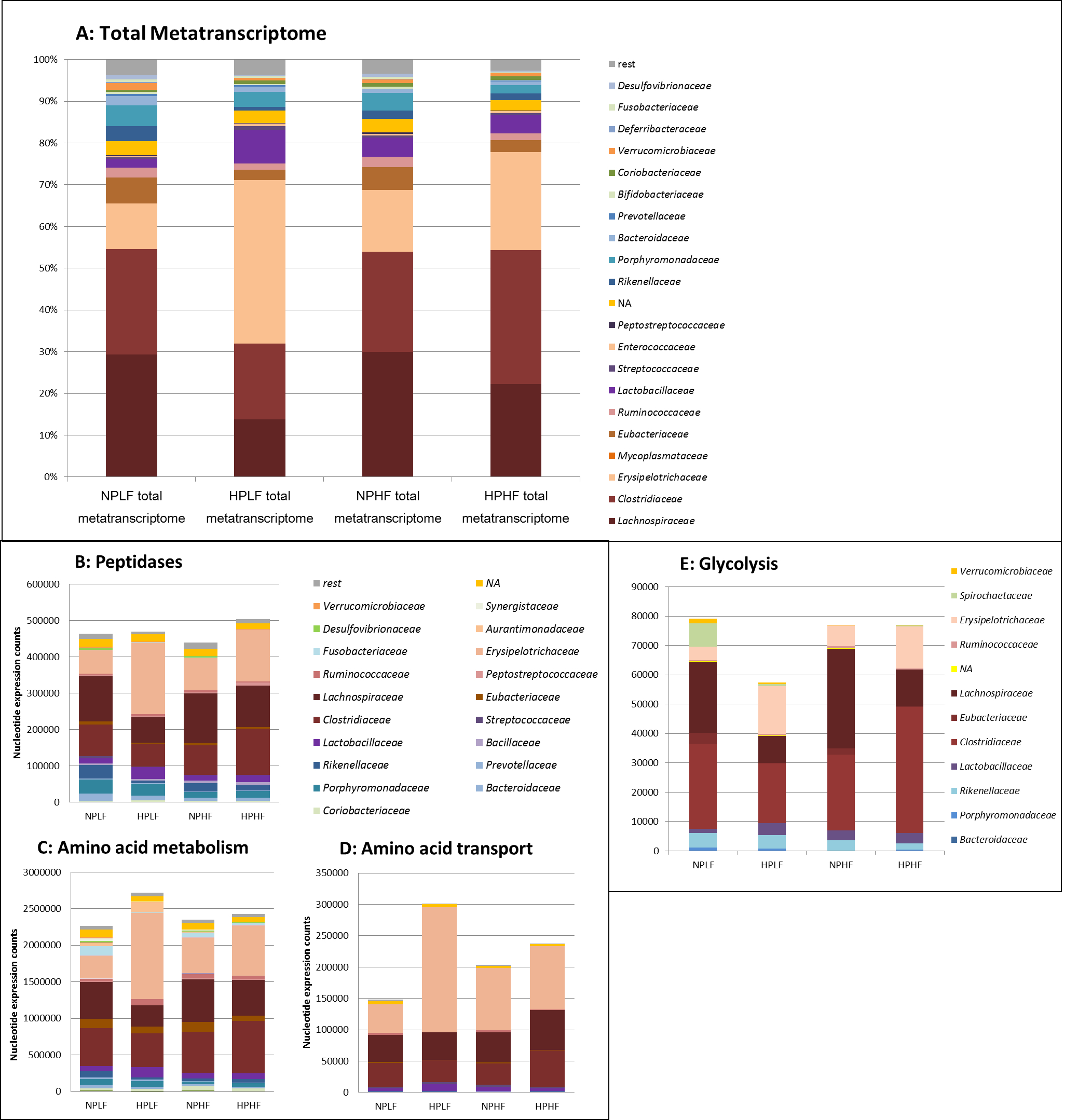

Supplement: S3 Fig — S3a Relative abundance of total metatranscriptome (activity) on family level. All the genes that were predicted with a KEGG orthology were accumulated and their taxonomic origin on family level is plotted here. Families with activity over 0.5% abundances in any of the conditions are plotted. S3bcd. Relative abundance of families expressing peptidases (b), amino acid metabolism related proteins (c) and amino acid transporters (d). All the genes that were predicted according to their KEGG orthology to belong to either peptidases, amino acid metabolism or amino acid transporters, were accumulated and their taxonomic origin on family level is plotted here. Families with activity over 0.5% abundances in any of the conditions are plotted. S3e. Expression level of 6-phosphofructokinase in the glycolysis pathway. This enzyme catalizes a step in the glycolytic pathway and its gene was transcribed at a lower level in the HPLF diet, which was mainly due to the decreased expression from Lachnospiraceae. (TIF) [file pone.0194066.s003.tif]

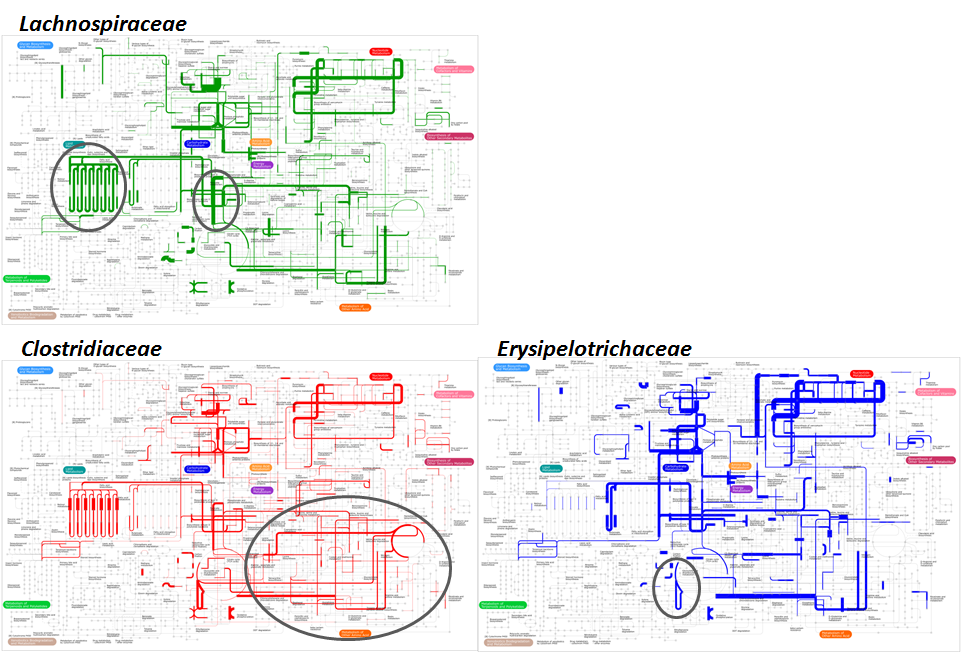

Supplement: S4 Fig — The expression patterns of all the KEGG numbers per individual family were plotted in with the iPATH software suite. In each of the microbial families unique expression patterns were found, which are indicated within the grey circles. Lachnospiraceae strongly expressed genes coding for enzymes involved in the conversion of phosphoenolpyruvate to oxaloacetate, and lipid biosynthesis activity. The Erysipelotrichaceae appeared to be much more focused on the conversion of malate, fumarate and succinate. The Clostridiaceae representatives were concluded to express both these features and a broad spectrum of pathways related to amino acid metabolism. (TIF) [file pone.0194066.s004.tif]
